# Associated risk factors for skin alterations in dairy cattle kept on small scale mountain farms

**DOI:** 10.1371/journal.pone.0285394

**Published:** 2023-08-08

**Authors:** Mousaab Alrhmoun, Thomas Zanon, Ioanna Poulopoulou, Katja Katzenberger, Matthias Gauly

**Affiliations:** Faculty of Science and Technology, Free University of Bolzano, Piazza Università 5, Bolzano, Italy; Universidade Federal de Mato Grosso do Sul, BRAZIL

## Abstract

The objective of this study was to determine the risk factors for skin alterations at herd and cow level on dairy farms with different housing systems in South Tyrol (Northern Italy). A cross-sectional study was conducted on 204 farms (111 free stalls and 93 tie stalls) from March to October 2019 assessing the level of animal welfare using resource-based and animal-based indicators. A total number of 1,891 dairy cows were evaluated, of which 43.5% were reared in tie stalls and 56.5% in free stalls. A logistic regression model identified the herd and cow level risks factors for neck and leg skin alterations in the two different systems. There was a higher prevalence for skin lesions on the neck (Odd Ratio (OR) = 2.36) and hock (OR = 2.82) for tie stalls. Irrespective of the housing system the soft-based stall mattresses had a lower prevalence for knee and hock lesions of 0.48 and 0.54, respectively, compared to wood base stalls for both knee (OR = 2.19) and hock (OR = 2.47) consecutively. The prevalence of skin alterations on the knee (OR = 0.42) and hock (OR = 0.33) decreased by the presence of sawdust as bedding material. Similarly, straw (OR = 0.61) and lime-straw-water bedding (OR = 0.59) reduced the prevalence for skin alterations on the hock. Access to pasture reduced the prevalence of skin alterations on the neck (OR = 0.34), the knee (OR = 0.77), and on the hock (OR = 0.46) regardless of the housing system. In conclusion, the assessment of risk factors of different skin alterations in different housing systems can contribute to the improvement of overall animal welfare in traditional small scale mountain dairy systems.

## Introduction

Despite the recent recommendations from the World Organization for Animal Health (OIE) [1] on appropriate housing standards for dairy cattle, tie stall barns, which are assumed to have some limitation in terms of expression of some natural behaviour patterns as well as higher prevalence for leg injuries and lesions than less-restrictive housing systems [2], are still the dominant housing system in many less favoured and marginalized areas such as the Italian Alps. In these areas environmental and topographical constraints, and the high investment costs make the transition from tie to free housing system a challenging task [2, 3]. In addition, the fact that tie stall barns, originally designed for smaller dual-purpose breeds, are now used for high yielding dairy animals such as Holstein Friesian or Brown Swiss, leads to an increased risk of developing skin alterations [4, 5]. The latter causes discomfort to the animals and if left untreated can lead to painful lesions that have been associated with a deterioration in animal welfare [6, 7]. Generally, skin alterations often occur around the hock and can range from a small area of hair loss to swelling and open wounds and in some cases can occupy the entire joint [8–10]. For instance, typical lesion sites in cattle, besides the knee and tarsus are the neck, fetlock, and the hip [7, 8, 11]. Skin alterations on the knee and hock are attributed to the protrusions that are present in the resting areas. When animals lie down, the soft tissue is compressed between these prominent bones and the bedding surface, resulting in an interruption of tissue perfusion which consequently leads to skin alterations and lesions [12]. Furthermore, other issues such as abrasiveness of the surfaces and microlesions which become infected also may play a role [13]. Among the risk factors contributing to the development of skin alterations on the limbs, in addition to housing type, are bedding material and the dimension of laying area [4, 8, 14, 15]. For instance, cows reared on concrete were more prone to swellings at the knee joints compared to cows reared on rubber mats [16], while cows housed on recycled sand were more likely to suffer from hair loss and swelling at the knee joints [15]. Damage to both front and hind legs was lower in straw-based systems compared to rubber mats or concrete lying surfaces [15, 17].

Although several studies have investigated the prevalence of skin lesions in tie stall [7, 18] or free stall housing systems [19, 20] to our knowledge, few studies have compared the two housing systems (tie stall and free stall) to determine the risk factor for skin alterations on mountain farms [21]. Therefore, since ensuring animal welfare is a prerequisite for the sustainability of traditional mountain livestock farming, the objectives of this study were to identify potential risk factors for herd and cow level skin alteration in tie and free stall housing systems on small scale mountain dairy farms to provide information for developing strategies to mitigate the occurrence of such incidents.

## Materials and methods

### Area of the study

The present study was conducted in the province of South Tyrol in the very northern part of Italy on the border to Austria and Switzerland from March 2019 to October 2019. Dairy production in South Tyrol is characterised by small scale farming with an average farm size of 15 dairy cows and producing 90,000 kg per farm and year. Due to small herds and limited space availability because of unfavourable topographical circumstances many farmers are still using tie stall housing systems.

### Data collection

This study was conducted as a part of WATS project “A Welfare Assessment Tool for South Tyrolean cattle farms.” The experimental and notification procedures were carried out in compliance with Directive 2010/63/EU. The participation of the farmers in the project was on a voluntary basis. In the present study 204 South Tyrolean farms (111 free stalls and 93 tie stalls) with a total number of 1,891 dairy cows (822 cows housed in tie-stalls (43.47%) and 1069 cows in free stalls (56.53%)) were considered. From the total number of animals evaluated, the number of animals and their distribution among the different breeds is as follows: Brown-Swiss; n = 645, Simmental; n = 512, Holstein-Friesian; n = 433, Tyrolean Grey; n = 204, Jersey; n = 37, Pinzgauer; n = 20 and others including crossbreeds (n = 40). Due to limited data availability Jersey, Pinzgauer and crossbreeds were not considered for statistical analysis. The assessment of animal welfare was performed by an expert (1 person) during farm visits according to the protocol for small scale mountain dairy farms proposed by [5], which comprise a total number of ten resource and animal-based indicators originally proposed by [22]. Inter-reliability testing of welfare outcome assessment was performed previously to ensure the quality of the data [23]. The detailed description of the protocol has been published in [5]. In addition, skin alterations on the neck, knee and hock were assessed according to the scoring system shown in Table 1 [5].

**Table 1:** Scoring criteria for alterations on three locations (neck, knee, and hock).

Lesion	Score 0	Score 1	Score 2	Score 3
**Hock**	no swelling	hair loss	swelling 1–2 cm high	lesion (Swelling > 2.5 cm high)
	no hair loss	no swelling	or broken skin, or/and	may have hair loss or
	no lesion	no lesion	hair loss	swelling <2.5cm or/and broken skin
**Knee**	no swelling	hair loss	Swelling 1–2 cm high	Lesion (Swelling > 2.5 cm high)
	no hair loss	no swelling	or broken skin, or/and	may have hair loss or
	no lesion	no lesion	hair loss	swelling <2.5cm or/and broken skin
**Neck**	no swelling	hair loss	Swelling 1–2 cm high	lesion (Swelling > 2.5 cm high)
	no hair loss	no swelling	or broken skin, or/and	may have hair loss or
	no lesion	no lesion	hair loss	swelling <2.5cm or/and broken skin

The expert observed the animal from the righthand side from 2 meters. Skin alterations were only considered if the surface of alteration was greater than a 10-euro cent coin.

Abnormal body condition score considered cows with a Body Condition Score lower than 3 (thin and very thin cows) [5].

Moreover, the number and dimensions of stalls/cubicles were recorded, as well as the quality of the flooring and bedding materials of the stalls, to obtain an insight into the space and comfort around the resting area.

### Statistical analysis

For data analysis, all analytical procedures were performed using SAS (version 9.4, SAS Institute Inc., Cary, C, USA). The experimental units were the cow level and farm level covariates. Descriptive investigation of potential risk factors was performed before entering the data into the models. The study population’s prevalence of hairless patches, swellings, broken skin was calculated at both the cow and the herd level. Confidence intervals (CI95%) of the population’s prevalence were estimated assuming a binomial distribution [16]. Given that there were 3 records for each individual cow (neck, knee and hock) and 10 cows in each herd, the cluster effect at the animal and herd level was included as a random effect using an alternating logistic regression with individuals nested within herd [24] by using the SAS statement Proc GENMODE. Using a binomial distribution with a log link function and Wald chi-square type 3 for significance testing, this method was used to investigate risk factors associated with the absence or presence of lesions on the neck, knee, and hock of dairy cattle in separate models. Lesions were classified as hairless, swollen, wound or open wound (1), or no lesions (0) [14]. Basic graphs were used to investigate linearity, and each variable was compared to each outcome separately. The general model used for estimating Logit (pi) was as follow:

$$Logit\;(pᵢ)=\beta^{0}+\beta^{1}X^{1}\;is+\ldots +\beta ₖ\;Xₖ\;ᵢₛ+Z\;farm(ᵢ)+Z\;individ(ₛ)$$

where: pi is the response variable, β0 is the intercept, (X1…Xk) the independent variable, β1X1 is + … + βkXk are fixed effects, and Z herd(i) + Z individ(s) are random effects due to herd and cow, respectively. Changes were made to categorized variables based on their percentiles or quartiles (10, 25, 50, 75, or 90%) for continuous variables that did not have a linear relationship with the outcome. Ordinary or hierarchical dummy variables were used to change other variables. First, each explanatory variable was tested separately, including random effects. Variables with a P-value <0.20 within this analysis were considered in the full model. A forward stepwise approach was used to construct the complete model. Each variable was included separately, any distortion or confounding could be observed. The model was run with and without the confounding variable to see how it affected the results. The best-fitting variables were chosen. Only one of the variables was included if there was a correlation between them. Deviance Δ was used to assess each model’s good fit.

## Results

### Prevalence of skin alterations

The degree of skin alteration observed at a farm level is shown in Table 2. Approximately 34.8% (71 farms) were assessed with moderate skin alterations (Score 1 according to Table 1) and 8.3% (17 farms) were assessed with severe skin lesions (Score 2 or 3 according to Table 1) (Table 2). The distribution of the prevalence of skin lesion types at farm level is highlighted in Table 3.

**Table 2:** Descriptive statistics for degree of skin alteration and type of skin alteration on farm level.

Level of skin alteration		
	Number of farms	Percent
No lesion	116	57%
Moderate*	71	34.8%
Severe^#^	17	8.3%

* Score 1

# Score 2 or 3

**Table 3:** Prevalence of skin alterations on the neck, knee, and hock in animals housed in different housing systems.

			Housing system			
	All cows (n = 1891)		Tie stall (TS)		Free stall (FS)	
Indicator [% of animals]	No. cows	%	No. cows	%	No. cows	%
Skin alteration on the neck^a^	451	23.85	372	45.26	79	7.39
Hair loss	233	12.33	180	21.87	53	4.99
Swelling	339	18.7	293	35.6	46	4.3
Lesion	13	0.72	6	0.73	7	0.65
Skin alteration at the knee^b^	870	46.01	506	61.56	364	34.05
Hair loss	756	39.98	411	53.17	345	33.24
Swelling	318	17.56	250	32.34	68	6.55
Lesion	4	0.22	1	0.13	3	0.29
Skin alteration at the hock^c^	809	42.78	573	65.33	272	25.44
Hair loss	802	42.41	533	69.76	269	25.82
Swelling	63	3.48	53	6.94	10	0.96
Lesion	11	0.61	5	0.65	6	0.58

*: p-value estimated by Chi-squared test

^a^ (TS: n = 822, FS: n = 1069)

^b^ (TS: n = 773, FS: n = 1038)

c (TS: n = 764; FS: n = 1042)

Overall, the prevalence of skin alterations on the neck, knee and hock in cows housed in tie stall systems was 45.26%, 61.56%, 65.33%, respectively, whereas the prevalence of skin alterations on the neck, knee and hock in cows housed in free stalls was significantly lower (p -value < .0001) with 7.39%, 34.05% and 25.44%, respectively (Table 3).

The risk factors significantly associated with the 3 locations with the highest prevalence, the neck, knee, and the hock, are depicted in Table 4. Regarding the model on neck alterations, 1306 cows had no alterations while 585 cows were assessed with hair loss, swelling, or a lesion. The covariates included in the model of the neck alterations model explained 48.3% of the variation. The risk factors for skin alterations on the neck were housing system (tie stall or free stall), access to pasture, breed, and body condition score.

**Table 4:** Risk factors in the final model for alterations on the neck, knee, and hock on 1891 cows in tie and free stall housing systems.

		Neck						Knee					Hock				
Factors	Classification	n	ß^1^	SE	OR^2^	CI^3^	*P-*value	ß	SE	OR	CI	*P-*value	ß	SE	OR	CI	*P-*value
Housing system	Tie stall	822	0.954	0.246	2.36	2.05–3.71	< .0001						1.594	0.143	2.82	1.98–3.05	< .0001
	Free stall	1069	-0.145	0.183	0.74	0.38–0.99	0.007						-0.84	0.637	0.44	0.36–0.66	0.014
Type of bedding	Sawdust	181						-0.987	0.18	0.42	0.33–0.68	0.003	-0.764	0.27	0.33	0.21–0.43	0.002
material	Straw	1534											-0.44	0.12	0.61	0.52–0.84	0.0006
	Lime-straw-water	29											-0.95	0.61	0.59	0.31–0.77	0.03
	Manure solids	40															
	No bedding	7						0.00	0.00	1.00			0.00	0.00	1.00		
Stall base	Rubber mats	53						0.638	0.27	2.13	1.83–2.77	0.04	-0.98	0.37	1.56	1.14–1.91	0.006
	Matten	872						-0.798	0.31	0.77	0.51–0.93	0.004					
	Wood	77						1.382	0.51	2.19	1.84–2.46	< .0005	1.44	0.62	2.47	1.75–3.14	0.0007
	Soft Mattresses	889						-1.48	0.39	0.48	0.22–0.84	< .0001	-0.91	0.11	0.54	0.44–0.72	ND^4^
Access to pasture	Yes	1338	-0.27	0.08	0.34	0.28–0.49	< .0001	-0.53	0.14	0.77	0.62–0.98	< .005	-0.88	0.2	0.46	0.32–0.59	0.0001
	No	553						0.99	0.17	1.43	1.1–1.89	0.02	1.42	0.35	2.83	1.84–3.41	< .0001
Breed	Brown-Swiss	645	0.88	0.31	1.98	1.15–2.24	0.04						-0.66	0.23	0.54	0.44–0.97	< .0001
	Simmental	512						0.67	0.18	1.83	1.51–2.72	< .0001	1.06	0.62	2.11	1.98–2.86	0.04
	Holstein-Friesian	433	0.39	0.07	1.62	1.29–1.93	0.006						-0.79	0.13	0.24	0.14–0.96	0.02
	Tyrolean Grey	204	-0.72	0.19	0.33	0.16–0.67	< .0001	-1.13	0.64	0.27	0.21–0.44	< .006	-0.33	0.08	0.41	0.23–0.52	0.04
Season of visit	Spring	923						-0.24	0.07	0.41	0.33–0.59	< .0001	-1.71	0.47	0.28	0.19–0.71	< .0001
	Summer	756	-0.55	0.09	0.31	0.15–0.50	< .072	-0.66	0.15	0.38	0.27–0.66	< .0001	-0.96	0.5	0.65	0.53–0.87	0.04
	Autumn	212	0.73	0.22	1.4	1.35–2.08	< .008						1.69	0.87	2.41	1.99–3.13	0.008
Body condition scores (BCS)	Normal body	1085															
	Abnormal body	806	1.96	0.74	2.21	1.58–2.57	0.004	0.93	0.08	1.13	1.03–1.89	0.02	1.34	0.61	1.77	1.22–2.47	0.003
Herd effect (α2)^5^			1.13	0.37	1.62	1.29–2.33	< .0001	1.81	0.57	2.41	1.58–3.39	< .0001	3.11	0.54	1.65	1.05–2.19	< .0001
Individual effect (α1)^6^			2.66	0.84	4.15	2.51–5.73	< .0001	3.41	0.22	15.16	8.32–17.89	< .0001	3.98	0.92	6.15	4.45–9.43	< .0001

^1^ Estimate.

^2^ Odds ratio.

^3^ Confidence interval, lower and upper limits.

^4^ ND = P > 0.05.

^5^α2 = random effect on herd level.

^6^α1 = random effect on individual cow level.

* Number of farms.

The model on knee alterations included 813 cows with no alterations and 1078 cows with hair loss, swelling, or a lesion. The covariates in the knee alterations model explained 64.2% of the variation. The risk factors for skin alterations on the knee were type of bedding material (sawdust), stall base, access to pasture, breed, season of visit, normal body condition score.

The model on hock alterations included 876 cows with no alterations and 1015 cows with hair loss, swelling, or a lesion. The covariates in the hock alterations model explained 42.8% of the variation. The risk factors for skin alterations on the hock were housing system, type of bedding material (sawdust, straw, lime- straw water), stall base (wood and rubber mats), access to pasture, breed, season of visit and abnormal body condition score.

The factors associated with the risk of skin alterations according to the housing system showed a similar OR for neck (OR = 2.36) and hock (OR = 2.82) alterations for the tie stall system while OR for the free stall system estimated at 0.74 and 0.44 respectively, while no alterations were observed on the knees.

**Fig 1:**
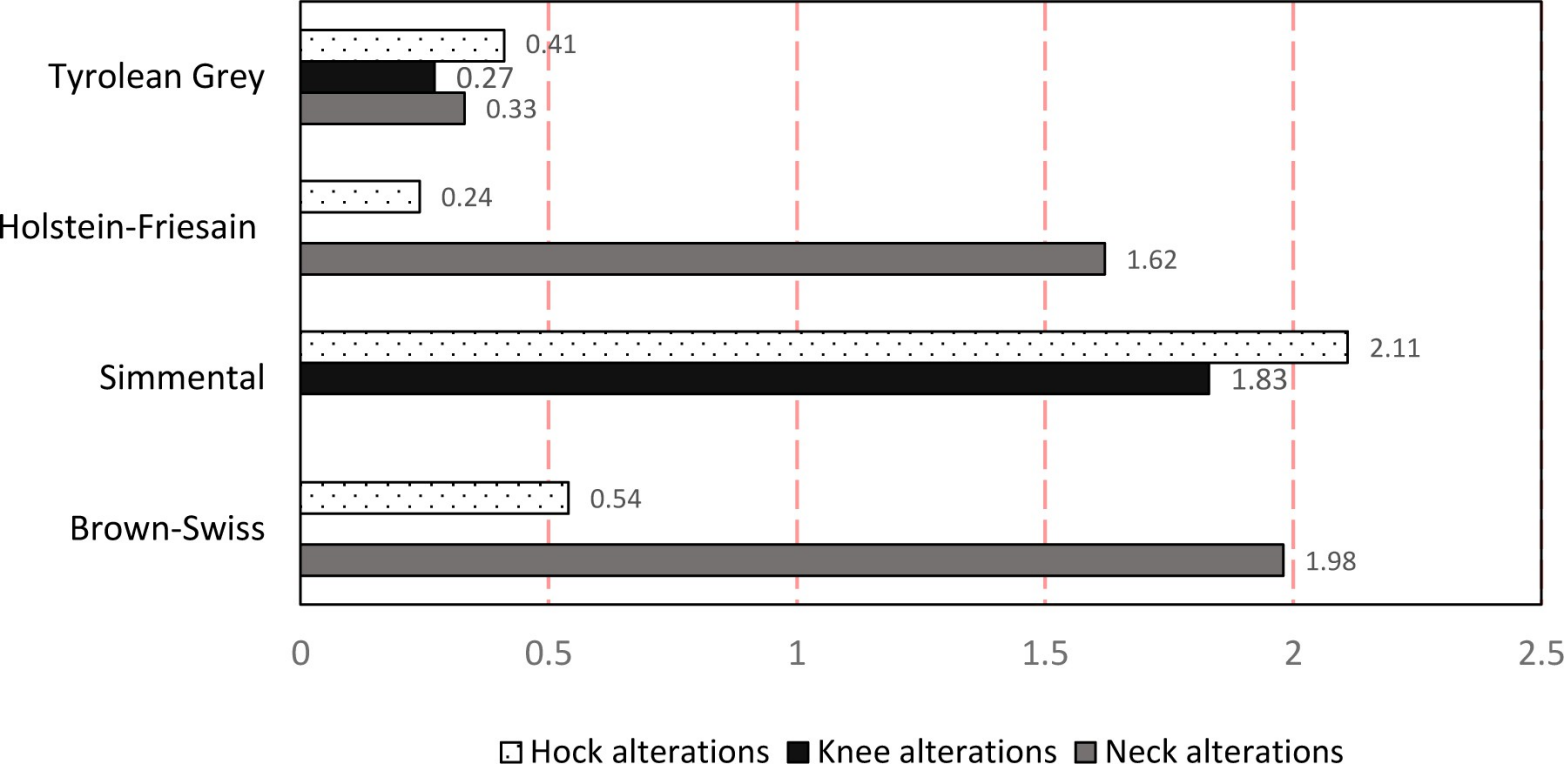
Breed OR alterations of the neck, hock, and knee. The OR are derived from the three separate and independent logistic models. P-values < 0.05 are circled (OR range from 0 to 2.5 with an increasing step of 0.025. p-value varies between 1.10 × 10 ^−4^ and 4.1 × 10 ^−2^, and y represent the variable in the three models.

In brief the factors that prevent the risk of developing skin alterations on the neck, knee and hock appear to be related to access to pasture, breed, and body condition score. The risk factors that further affected skin alterations on legs (both knee and hock) were associated to the type of bedding material, stall base and season of visit. Increased ORs for the development of neck alterations were observed for Brown Swiss (OR = 1.98) and Holstein Friesian (OR = 1.62) while Simmental cows showed increased ORs for knee (OR = 1.83) and hock (OR = 2.11) skin alterations. Tyrolean Grey showed reduced ORs for skin alterations on the neck, knee and hock (Fig 1).

When investigating the individual effect of the cows, the model relating knee and hock alterations revealed an OR of 15.16 and 6.15 which indicated a large individual correlation between the knee and hock. This effect was less extreme regarding neck alterations (OR = 4.15). The herd effect revealed an OR of 2.41, indicating an individual correlation between cows within the herd regarding knee lesions. This effect was lower for neck lesions (OR = 1.62).

## Discussion

Our attempt to identify potential risk factors for skin alterations in small scale mountain dairy farms revealed that the housing system significantly influenced the occurrence of skin alterations on the neck, the knee, and hock, as the risk for skin alterations on the neck and hock were lower when cows were housed in free stalls (Table 4). Similar results were previously published in [25]. Moreover, the present results suggest that risk factors associated with skin lesions vary by lesion site, supporting the results of [12, 16]. Following previous work, we analysed the neck, knee, and hock separately in the model and managed the repeated measures by including "individual" as a random effect in the model. In this way, correlations could be demonstrated between findings in the neck, knees, and hock, and not just fixed effects. The risk of such skin alterations was higher when cows were housed in tie stalls (Table 4). Significant neck lesions in tie stalls were also reported by [12] who associated the rail high with 70% less neck lesions compared to the mid tie-rail (116–132 cm). Similarly, the risk of skin alterations on the hock was significantly affected by the housing system and was higher when cows were housed in tie stalls (Table 4) [26].

In addition, the type of bedding material such as straw, sawdust, and lime-straw-water, was shown to be a significant risk factor for reducing skin alterations on the hock (Table 4) which is line with [7, 27]. The latter observed a reduced prevalence of skin alterations on the hock in dairy cows when sawdust (OR = 0.19), sand (OR = 0.33) or chopped straw (OR = 0.85) was used as bedding material. Likewise, [7] observed a reduced risk of severe skin lesions such as ulcers when deep straw was used as bedding material which is in line with our findings (Table 4). Regarding the stall base, rubber mats increased the risk for skin alterations at the knee and at the hock (Table 4) [7, 15, 19, 27, 28]. Similarly, for both hock and knee, the wooden lying area increased the risk for skin alterations, which highlights the importance of soft bedding material and lying surface to improve rest and reduce skin alterations and lesions in dairy cows as already stated in previous literature [7, 29–32]. In contrast to the results of the present study, [28, 33] found that heifers kept in mattresses, had significantly worse hock lesions compared to heifers kept on rubber mats. Overall, the presence of hard base in the stall increases the pressure on the hock when cows are lying down and consequently increases the risk of reduced circulation and subsequent tissue damage [19] (Table 4). The latter is further revealed in [34] which stated that soft lying mats are less favourable than straw bedding in terms of tarsal injuries.

Furthermore, our results (Table 4) highlight that the risk of neck, knee and hock alterations can be reduced when cows have access to pasture, while [35] also report the beneficial effect of access to pasture on the overall animal health and welfare [36, 37]. In a similar study, [25] observed that access to regular outdoor exercise in cows kept in tie stalls resulted in a lower prevalence of skin injuries around the hock throughout the year, compared to cows kept in tie stalls with limited outdoor exercise during the winter. Moreover, [37, 38] observed a reduction in the likelihood of hock alterations such as hair loss, swellings, or lesions in dairy cows with increasing hours of access to pasture.

In addition, results of the present study consistently revealed Tyrolean Grey to have a lower risk of skin alterations compared to Brown Swiss, Simmental, and Holstein Friesian. This is in line with findings reported in [4, 21, 39] who highlighted that breed has a significant effect on the welfare of dairy cows housed in traditional tie stalls in the Italian Alps, as local dual-purpose breeds, seem to be better adapted to the local conditions. The latter is confirmed by [40, 41] who described generally a low prevalence for health issues for local dual-purpose breeds and therefore lower resulting veterinary costs [42].

Abnormal body condition (thin and very thin) increased the OR for skin alterations at all three sites (neck, knee, and hock). This result is supported by [20, 32] who described an association of BCS with the prevalence of skin alteration in dairy cows. Lastly, the risks for neck, knee and hock alterations were reduced in summer and spring and increased in autumn (Table 4) which might be attributed to access to pastures during vegetation period, which was previously shown to have a beneficial effect on animal health and welfare [3, 35].

Finally, the association between the risk factors and skin alterations, identified here, do not prove causality and, therefore, some of the risk factors that were detected may be features of other variables such as the analysis as lameness, days in milk, and environmental conditions that were not considered for the present assessment. Although only farms that agreed voluntarily in participating in the study were considered for the assessment and therefore might cause some unavoidable bias, our results offer an important new insight into the risk factors associated with different skin lesions on the neck, the knee, and the hock in cows housed in traditional small scale mountain dairy systems. The latter should contribute to improving the overall animal welfare in such systems which in terms of management and farm structure largely differ from large-scale dairy farms in lowlands. The 95% CI determined for each skin alteration, was narrow, indicating good reliability of the current results and sufficient sample size at cow level, ensuring comparability and data quality.

## Conclusions

The present study aimed to identify potential risk factors for herd and cow level skin alteration in tie and free stall housing systems on small scale mountain dairy farms. The risk factors identified for skin alterations were mainly related to the type of housing systems, the conformation of stall base, the type of bedding material selected, and access to pasture. Future studies should consider our findings for developing causal cow-level studies for deepen the knowledge on animal health and welfare in small-scale mountain dairy farms. The latter differ to a large extent from large dairy farms in the lowlands in terms of management, facilities, and barn design, which is why a differentiated view of these farms is necessary.

## Supporting information

S1 File Distribution of the variables that were assessed in 3 independent models (logistic regression), but not significantly associated with the model for the neck, knee, or hock alterations (n = 1891 individual in each model). (PDF)
