# Supplementary material for: Associated risk factors for skin alterations in dairy cattle kept on small scale mountain farms
Source: PLoS One. 2023 Aug 8;18(8):e0285394. doi: 10.1371/journal.pone.0285394 (PMC10409271; doi:10.1371/journal.pone.0285394)
Supplement: S1 File — (PDF) [file pone.0285394.s001.pdf]

Supporting information

SM1. Distribution of the variables that were assessed in 3 independent models (logistic regression), but not significantly associated with the model for the neck, knee, or hock alterations (n =1891 individual in each model).

Variable	Classification	cows, n (%)	Mean	SD
**Water flow rate per minute**				
	> 20 liters	75, (3.97)	21.8	± 7.9
	< 10 liters	623, (32.9)	7.4	± 2.5
**Herd size**				
Number of dairy cows	≤10 cows	520, (27.49) (51 farms)	8	± 3.7
	>10 cows	1371, (72.5) (153 farms)	16	± 5.4
**Dimensions of stalls/cubicles**				
length	metric level (m)	1891	1.94	± 0.84
width	metric level (m)	1891	1.2	± 0.43
